# The Global Prevalence of *Schistosoma mansoni*, *S. japonicum*, and *S. haematobium* in Pregnant Women: A Systematic Review and Meta-Analysis

**DOI:** 10.3390/tropicalmed7110354

**Published:** 2022-11-04

**Authors:** Leslie Faye T. Cando, Glenmarie Angelica S. Perias, Ourlad Alzeus G. Tantengco, Micah D. Dispo, Jeremy A. Ceriales, Mark John G. Girasol, Lydia R. Leonardo, Ian Kim B. Tabios

**Affiliations:** 1 College of Medicine, University of the Philippines Manila, Manila 1000, Philippines; 2 Department of Epidemiology and Biostatistics, College of Public Health, University of the Philippines Manila, Manila 1000, Philippines; 3 Office of Research Coordination, University of the East, Manila 1008, Philippines; 4 Institute of Biology, College of Science, University of the Philippines Diliman, Manila 1101, Philippines; 5 Institute of Human Genetics, National Institutes of Health, University of the Philippines Manila, Manila 1000, Philippines

**Keywords:** parasitic infection, pregnancy, prevalence, Schistosoma haematobium, Schistosoma japonicum, Schistosoma mansoni

## Abstract

Schistosomiasis is a neglected tropical disease affecting 40 million women of childbearing age worldwide. Its global disease prevalence among pregnant women is still unknown. This meta-analysis determined the pooled prevalence of schistosomiasis among pregnant women globally. Additionally, this study also determined the pooled prevalence based on infection intensity based on eggs per gram. Observational studies on the prevalence of schistosomiasis among pregnant patients were obtained from Medline, Scopus, and CINAHL from January 2001 until August 2020. A review of titles and abstracts was done independently by six reviewers. The quality of the included studies was assessed using the Newcastle–Ottawa Scale for case–control, cohort, and cross-sectional studies. A total of 27 studies were included in the meta-analysis and meta-regression. The pooled prevalence of *S. haematobium* was 13.44 (CI: 8.90–19.80) per 100 observations, while the pooled prevalence of *S. mansoni* was 12.18 (CI: 4.47–29.12) per 100 observations. The prevalence of *S. japonicum* infection in one study was 53.54 (CI: 43.23–63.62) per 100 observations. Our results showed a prevailing health problem of schistosomiasis during pregnancy in various countries worldwide. This strengthens the need to conduct more schistosomiasis research, prevention, and control programs in pregnant women.

## 1. Introduction

Schistosomiasis is a neglected tropical disease caused by parasitic infection of the genus *Schistosoma* with the most common disease-causing species being *Schistosoma haematobium, Schistosoma mansoni*, and *Schistosoma japonicum* [1]. In 2018, it was estimated to affect at least 290.8 million people, claiming 24,068 lives globally [1,2]. It is also highly debilitating, leading to an estimated loss of 1.43 million all-age disability-adjusted life years [3]. The infection is widespread in the tropics and subtropics, with considerable morbidity in parts of the Middle East, South America, Southeast Asia, and sub-Saharan Africa. It is also linked to poverty, leading to chronic ill health [4]. 

Despite these, its disease burden remains underestimated and has resulted in insufficient resource allocation to schistosomiasis research and control [4]. One population group with a scarcity of data on Schistosoma infections is the population of pregnant women. Currently, schistosomes are estimated to infect 40 million women of childbearing age, but little is known about the prevalence and schistosome-associated morbidity in pregnant women and their newborn [5]. The effects of the disease in pregnant women are thought to be greater as infection will affect the mother and the growing fetus [4]. Although it is not well understood due to the paucity of studies and conflicting results, the proposed mechanisms of schistosomiasis-mediated adverse birth outcomes are placental inflammation, maternal and fetal iron deficiency due to extracorporeal iron loss, anemia of inflammation, and anorexia due to proinflammatory cytokines. These may lead to problems such as intrauterine growth restriction [5]. Additionally, pregnancy is thought to exacerbate *Schistosoma*-induced pathology due to the immunological shift to Th2 during pregnancy because of progesterone and placental products [6,7]. It has also been hypothesized that maternal and childhood infections reduce the effectiveness of childhood immunizations and increase susceptibility to viral and bacterial diseases [4]. However, these need to be verified and studied more thoroughly.

Initially, there was a lack of adequate and well-controlled studies that demonstrated the safety of using praziquantel as a treatment for schistosomiasis in pregnancy. This became a significant challenge for implementing this treatment program among pregnant women. Although the WHO published a report in 2003 stating that all schistosome-infected pregnant and breastfeeding women be considered high-risk groups and be offered treatment with praziquantel either individually or during treatment campaigns, the lack of sufficient safety data from controlled trials made many countries reluctant to follow this recommendation [8]. This has been improving slowly since 2006, when positive results of two randomized clinical trials encouraged more countries to follow the WHO recommendation [9,10]. In 2011, the World Health Organization advocated for the disease control of schistosomiasis by 2020 and its elimination as a global health problem by 2025. It defines morbidity control as having a prevalence of heavy-intensity infection (PHI) that is below 5% in all sentinel sites and elimination as a public health problem as having a prevalence of heavy-intensity infection below 1% in all sentinel sites [11]. The intensity of infection is the number of schistosomes infecting an individual and is expressed by the number of helminth eggs excreted per gram of feces and by the number of eggs seen per 10 mL of urine. This is used as a marker for morbidity as chronic sequelae are suggested to be associated with cumulative exposure to schistosomes [12]. This may be because eggs excreted by adult schistosome worms need to leave the body in via the fecal route else they become trapped in nearby tissues. This induces a distinct immune-mediated granulomatous response that leads to a range of problems not limited to anemia, impaired cognition, severe hepatosplenism, periportal fibrosis, and urogenital inflammation. However, there is a lack of studies associating the infection intensity with disease burden in pregnant women [13].

As of 2020, some countries still have not yet adopted the mass drug administration policy [12]. Additionally, even in countries that adopt this policy, pregnant women are still excluded because implementers are unaware of the changes. Such exclusion causes millions of women of reproductive age to possibly miss treatment for many years during repeated cycles of pregnancy and lactation [14]. Therefore, it is imperative to advocate for more research in maternal schistosomiasis to help provide policymakers with adequate information for better control strategies in pregnant women to prevent these outcomes and lessen the overall prevalence of schistosomiasis [4]. Identifying and quantifying the effect of infection on the pregnant population may also play a part in improving birth outcomes in endemic areas [14].

## 2. Materials and Methods

### 2.1. Study Selection

**Figure 1:**
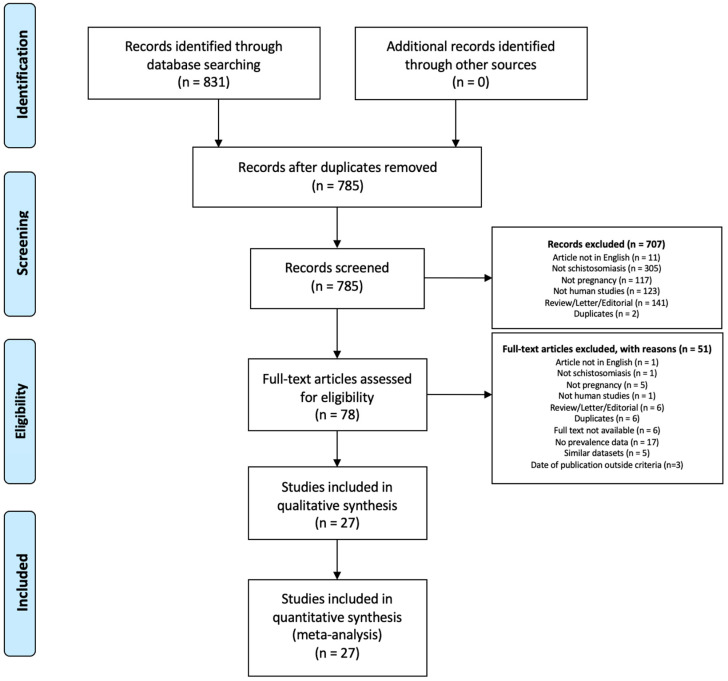
Search strategy and study selection for studies on the prevalence of schistosomiasis in pregnant patients.

The systematic review and meta-analysis were conducted based on the PRISMA flow diagram (Figure 1). The protocol was registered in PROSPERO (CRD42020212181). A systematic review of the literature was performed in three electronic databases: OVID Medline, Scopus, and CINAHL, from January 2001 to August 2020. Search strings were provided in the supplementary material (Supplementary File S1). Articles included in the study were restricted to those written in English. The most recent or complete study was used for articles with overlapping data and by the same authors. Review articles, letters to the editor, comments, case reports, and preclinical studies were excluded.

### 2.2. Inclusion and Exclusion Criteria

Eligible studies were included based on the following inclusion criteria: (i) Cross-sectional and cohort studies that determined the prevalence of schistosomiasis (*S. japonicum*, *S. haematobium*, and *S. mansoni*) in pregnant women; (ii) schistosomiasis was diagnosed using molecular, parasitological, rapid diagnostic test (urine CCA), serological, and ultrasonographic methods; (iii) sufficient data to calculate the prevalence of schistosomiasis. The exclusion criteria included schistosomiasis diagnosed by clinical questionnaires without confirmation through standard diagnostic methods for schistosomiasis.

### 2.3. Data Extraction

A review of titles and abstracts was done independently by six reviewers. Full-text articles were retrieved for all the eligible studies. Full-text articles were evaluated independently by six reviewers (OAT, IKT, MG, LFC, JC, and GAP). All irrelevant articles were excluded, with recordkeeping of the reasons for exclusion. The following data were collected from each included study: first author, year of publication, country of origin, study design, sample size, prevalence or incidence of schistosomiasis, detection methods, and source of specimens.

### 2.4. Assessment of Study Quality

The quality of the included studies was assessed using the Newcastle–Ottawa Scale for case–control, cohort, and cross-sectional studies. The scale is composed of eight questions covering three domains: (1) selection of study groups (four points for case–control and cohort studies, and five points for cross-sectional studies); (2) comparability of groups (two points); and (3) ascertainment of exposure and outcomes (three points). The scale assigns a maximum score of nine for case–control and cohort studies and a maximum score of ten for cross-sectional studies which represent a high-quality study [15,16].

### 2.5. Statistical Analysis

All analyses were done in R v.4.1,1 (R Foundation for Statistical Computing, Vienna, Austria) using the ‘meta’ package (version 4.19-0) [17]. The ‘metaprop’ function was used to conduct the meta-analysis of single proportions to obtain the pooled prevalence per species of *Schistosoma*. A random-effects model was employed through generalized linear mixed models (GLMM) with logit transformation for pooling studies. GLMM, particularly a random intercept logistic regression model, is a one-step approach in pooling proportions, which is generally less biased compared to two-step approaches [17,18]. The 95% confidence intervals for the individual studies were calculated using the Clopper–Pearson method, also known as the exact binomial method, which provides more conservative estimates [19]. Heterogeneity was evaluated using the Cochran’s Q test, the T^2^, and the I^2^ statistics. A statistically significant Cochran’s Q test at α = 0.05 indicates evidence of true heterogeneity of effect sizes between studies. The T^2^ statistic is the estimate of between-studies variance obtained in this study through the maximum likelihood method. The I^2^ statistic describes the percentage of total variability due to between-study variability, wherein an I^2^ value of 75% or higher shall be considered to indicate substantial heterogeneity.

Potential sources of heterogeneity were explored through meta-regression using the ‘metareg’ function. Univariable meta-regression was performed on species with at least ten studies [20]. The covariates considered for meta-regression were study design, sample size, specimen for diagnosis, and timing of study relative to approval of praziquantel mass drug administration for pregnant women, all measured as dichotomous variables. The regression coefficient estimates the difference in logit prevalence compared to the reference group and was considered statistically significant at α = 0.05. Publication bias was evaluated through the Egger’s test, wherein a statistically significant intercept at α = 0.05 indicated evidence of funnel plot asymmetry.

## 3. Results

### 3.1. Study Selection

In this study, 831 records were selected from three databases of published literature. After the removal of duplicates, 785 unique records remained. After the selection process, 27 studies were included in the qualitative synthesis, meta-analysis, and meta-regression (Figure 1).

### 3.2. Study Characteristics

Most studies (26/27) were from African countries and only one was from the Philippines (Supplementary Table S1). Seven (7) were cohort studies and 20 cross-sectional. A steady increase was noted from 2005 until 2020. The most common species of *Schistosoma* diagnosed among pregnant patients was *S. haematobium* (20/27), followed by *S. mansoni* (8/27), and *S. japonicum* (1/27). Four (4) studies detected both *S. haematobium* and *S. mansoni* (Supplementary Table S1).

The most used sample for diagnosing schistosomiasis among pregnant patients was urine sample alone (15/27), followed by stool sample alone (6/27). Several studies used both stool and urine samples (6/27). In terms of diagnosing schistosomiasis, the most used was urine filtration, centrifugation, and microscopy (17/27) and stool Kato Katz (8/27) (Supplementary Table S1).

Most cross-sectional studies were assessed to be of good quality with an average score of 7.80 based on the Newcastle–Ottawa Scale (Supplementary Table S2). The cohort studies were similarly assessed as good quality with an average score of 6.86 (Supplementary Table S3).

### 3.3. Synthesis of Meta-Analysis Results

**Figure 2:**
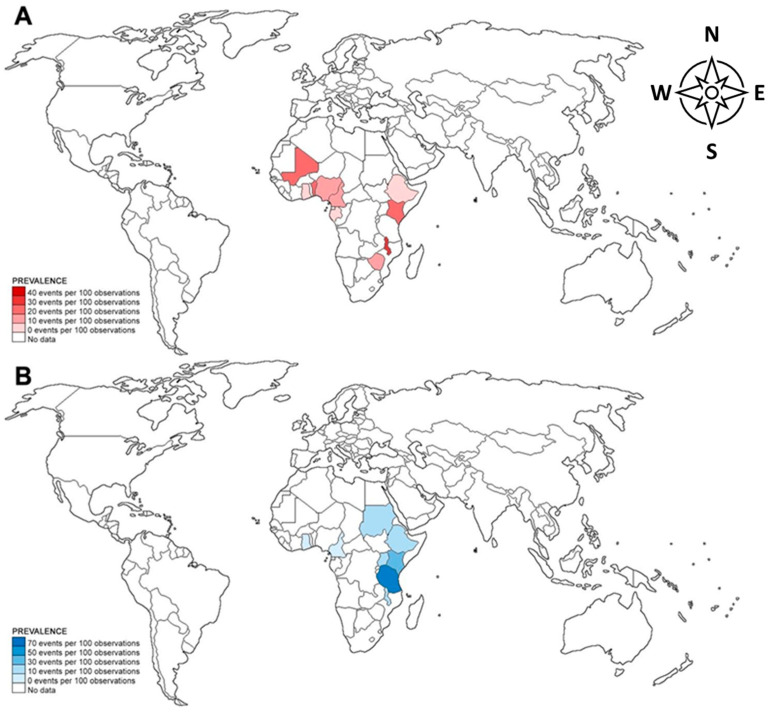
Pooled prevalence of *S. haematobium* (**A**) and *S. mansoni* (**B**) infection among pregnant patients in different countries. Borders of countries on the map do not imply any political statement.

In terms of geographic distribution, the prevalence of *S. haematobium* and *S. mansoni* was highest among pregnant women in African countries while *S. japonicum* was only reported in pregnant women from the Philippines. *S. haematobium* infection among pregnant women was highest in Malawi (32.31%), while *S. mansoni* infection was highest in Tanzania (63.48%) (Figure 2).

**Figure 3:**
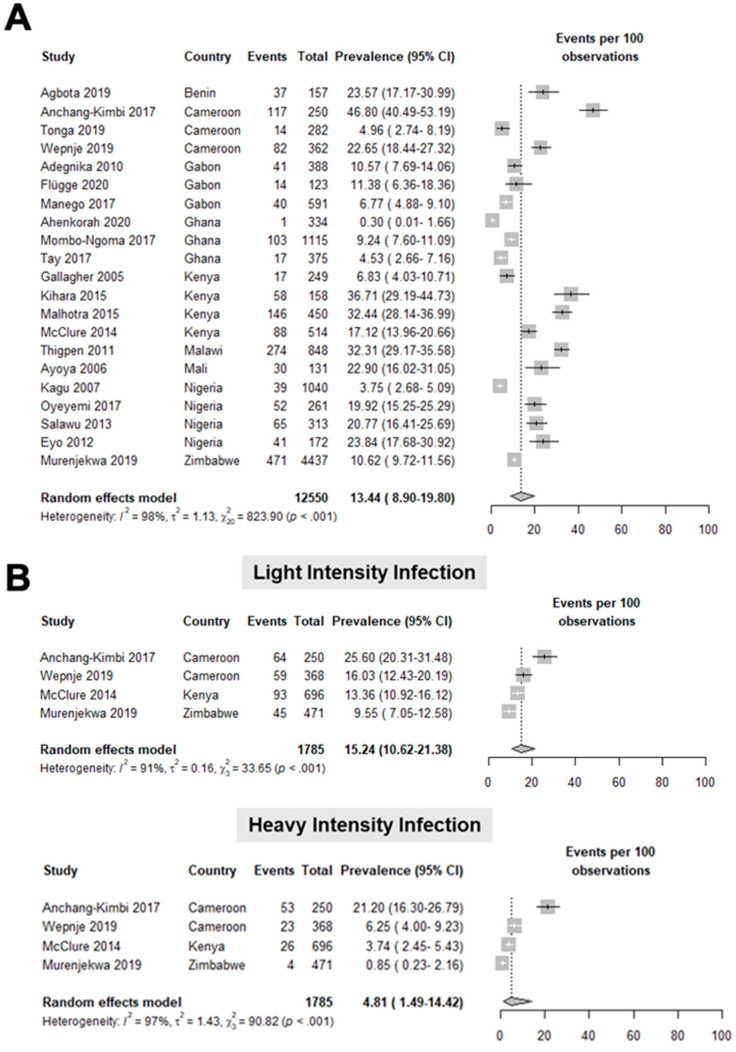
Forest plot for the (**A**) general prevalence (events per 100 observations) and (**B**) prevalence per intensity of infection of *S. haematobium* in pregnant patients worldwide [21,22,23,24,25,26,27,28,29,30,31,32,33,34,35,36,37,38,39].

Based on 21 studies [21,22,23,24,25,26,27,28,29,30,31,32,33,34,35,36,37,38,39] involving 12,550 pregnant women, the pooled prevalence of *S. haematobium* infection was 13.44 (8.90–19.80) per 100 observations (Figure 3A). The studies included in this meta-analysis had a high heterogeneity, as shown by a T^2^ value of 1.13, an I^2^ value of 98%, and a statistically significant Q test (*p* < 0.001). Upon classifying the intensity of *S. haematobium* infections, it was noted that light-intensity infection had a pooled prevalence of 15.24 (10.62–21.38) per 100 observations, while the heavy-intensity infection was 4.81 (1.49–14.42) per 100 observations (Figure 3B; Supplementary Table S4). 

Based on one study [40] with 99 pregnant women, the prevalence of *S. japonicum* among pregnant women in the Philippines was 53.54%. We then performed a subgroup analysis to determine the prevalence of *S. japonicum* infection based on the intensity of infection. The prevalence of light-intensity *S. japonicum* infection was 42.42 (32.55–52.77) per 100 observations, while the moderate-intensity infection was 11.11 (5.68–19.01) per 100 observations (Supplementary Table S4).

**Figure 4:**
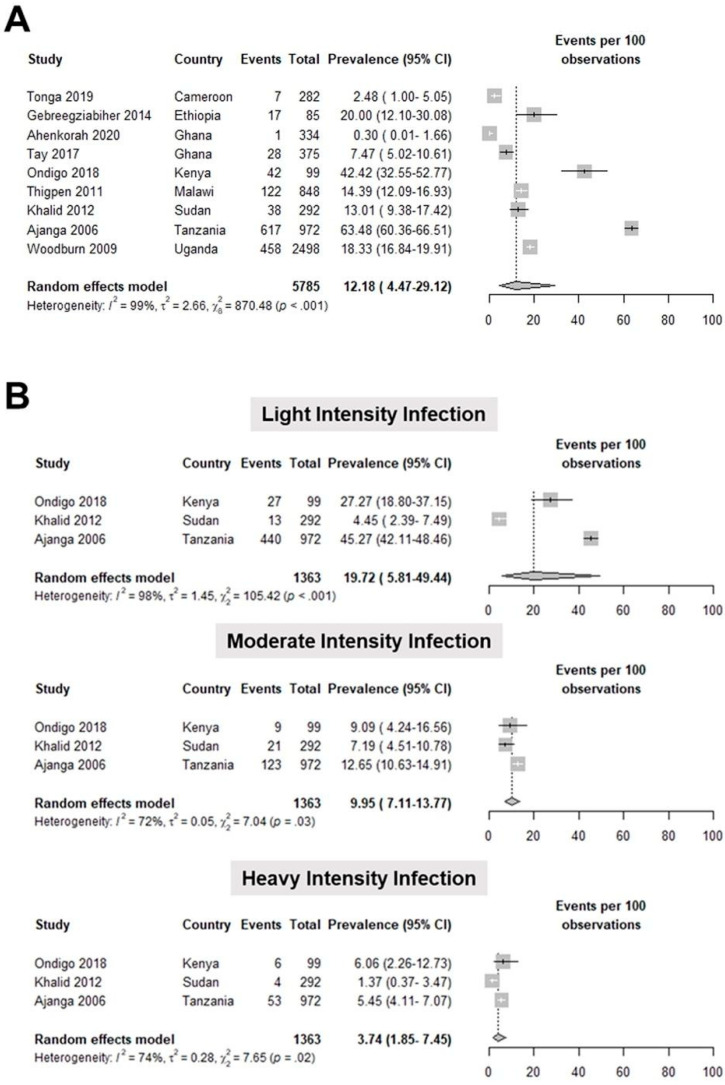
Forest plot for the (**A**) general prevalence (events per 100 observations) and (**B**) prevalence per intensity of infection of *S. mansoni* in pregnant patients worldwide [28,29,30,32,41,42,43,44,45].

Meta-analysis of nine studies [28,29,30,32,41,42,43,44,45] involving 5785 pregnant women showed that the pooled prevalence of *S. mansoni* infection was 12.18 (4.47–29.12) per 100 observations (Figure 4A). The studies included in this meta-analysis had a high heterogeneity, as shown by a T^2^ value of 2.66, an I^2^ value of 99%, and a statistically significant Q test (*p* < 0.001). We further classified the intensity of *S. mansoni* infection and determined the prevalence of light-intensity infection to be 19.72 (5.81–49.44) per 100 observations. The pooled prevalence of moderate-intensity infection was 9.95 (7.11–13.37) per 100 observations, while heavy intensity-infection was 3.74 (1.85–7.45) per 100 observations (Figure 4B; Supplementary Table S4).

### 3.4. Meta-Regression

Meta-regression was performed to investigate potential contributing factors to the heterogeneity of studies on the logit prevalence of *S. haematobium* (Table 1) among pregnant women, namely, study design, publication of the study before or after the approval of praziquantel mass drug administration for pregnant women, sample size, and specimen used for diagnosing schistosomiasis. Univariable analysis for *S. haematobium* infection revealed insufficient evidence of a linear relationship between the tested covariates and the logit prevalence (Table 1). The number of studies for *S. haematobium* was inadequate to conduct multivariable meta-regression with all covariates included in the model.

### 3.5. Publication Bias

Results of classical tests of asymmetry, particularly the Egger’s test, revealed no sufficient evidence of publication bias or small-study effects for *S. haematobium* (*p* = 0.76) and *S. mansoni* (*p* = 0.40) (Supplementary Table S5).

## 4. Discussion

A total of 27 studies were included for the qualitative synthesis, meta-analysis, and meta-regression for the prevalence of schistosomiasis in pregnancy. Only studies done from 2001 were included because it was then that the WHO promoted praziquantel for mass drug administration through the Fifty-fourth World Health Assembly in resolution WHA54.19 [46]. Data before this year may not reflect the current global efforts against schistosomiasis and may skew the current prevalence.

The most common species diagnosed among pregnant patients was *S. hematobium* with a pooled prevalence of 13.44%. This was followed by *S. mansoni* with a pooled prevalence of 12.18%; finally, *S. japonicum*, found only in one study, has a prevalence of 53.54%. A similar meta-analysis of schistosomiasis in pregnancy, which included 32 studies in Africa, had a pooled prevalence estimate of 13.2% [47]. The availability of schistosomiasis prevalence classified according to infection intensity based on eggs per gram (EPG) of stool for *S. mansoni* and eggs per 10 mL urine for *S. haematobium* in several studies allowed further analysis on this parameter [25,31,35,40,41,43,44,48]. According to WHO, *S. haematobium* infection intensity is classified as light infection if with 1–49 eggs per 10 mL of urine and heavy infection if with ≥50 eggs per 10 mL of urine. For *S. mansoni*, it is classified according to the number of eggs per gram of stool: 1–99 EPG for light infection, 100–399 EPG for moderate infection, and ≥400 EPG for heavy infection [49]. It can be observed that there is a decreasing trend in both *S. mansoni* and *S. haematobium* in prevalence as the intensity increases. The analysis for *S. haematobium* showed high heterogeneity, but none of the potential sources included in the analysis showed sufficient evidence of a linear relationship to the logit prevalence.

There are limited studies comparing the prevalence of schistosomiasis in pregnant and non-pregnant women. One study in Kenya reported that the prevalence of *S. haematobium* in non-pregnant women was 30% compared to 36.94% in pregnant women [39]. The non-pregnant women had an intensity of 40.88 eggs/10 mL urine compared to 51.21 eggs/10 mL urine in pregnant women. However, the prevalence and intensity of infection between the two populations are not statistically different. In addition, several other studies reported the prevalence of schistosomiasis in women of reproductive age, although not specifying the exclusion of pregnant women in their studies. These ranged from 4.5% in Tanzania to 62% in Uganda [50,51,52,53,54]. With these limited findings, it may be possible to suspect that maternal schistosomiasis is either only carried over from infection before their pregnancy or that the immunologic changes of pregnancy may have increased the susceptibility of pregnant patients to schistosomiasis [55].

Pregnancy causes significant changes in the body’s immune response. Generally, pregnancy causes a bias toward an anti-inflammatory environment to avoid fetal rejection—affecting disease pathogenesis during pregnancy. Several proteins and hormones are altered during pregnancy to favor the anti-inflammatory milieu. For example, the activity of steroids that suppress the transcriptional regulation of inflammatory cytokines such as IFN-γ, which has essential anti-viral and anti-parasitic properties, is suppressed. This contributes to significantly worse outcomes from infectious diseases during pregnancy [56]. Specifically, schistosomiasis infection during pregnancy is exacerbated due to an immunologic shift to Th2, probably through the up-regulation of parasite-specific IL-4 due to progesterone and placental products [6].

Accounting for the anti-inflammatory shift during pregnancy and the poorly understood sequelae of maternal schistosomiasis in the offspring, it is crucial to decrease the disease burden in pregnant women. In addition to these, schistosomiasis infection may also be a risk factor for several diseases, including human immunodeficiency syndrome (HIV) and human papillomavirus (HPV) infection [57,58]. Praziquantel (PZQ) is the drug of choice against all schistosome infections with its reliable therapeutic effectiveness, even on severe morbidity in endemic areas [14]. A randomized clinical trial on PZQ in pregnant women with low-intensity infection showed that the treatment effectively reduced maternal infection. Results show a significantly higher proportion of women with more than 90% reduction in eggs per gram compared to placebo and a cure rate of 83.7% [10]. Another study showed a decrease in *S. mansoni* infection from 16.7% to 4.8% at delivery with praziquantel treatment [9]. These support the efficacy of PZQ in reducing the schistosomiasis burden in pregnant women, despite the suppression of antibody levels against schistosomes and PZQ-induced boosts in antibody levels [59]. Before the treatment of schistosomiasis in pregnancy was endorsed by the WHO, PZQ treatment was avoided during pregnancy and lactation due to the lack of safety studies [8]. This served as a significant barrier to treating schistosomiasis in pregnant women. Despite the WHO recommendation to include pregnant women in PZQ treatment, there are still countries that have not adopted this due to the lack of relevant information and a failure to carry out appropriate risk: benefit analyses about the use of anthelminthic drugs during pregnancy and lactation; and for the countries that have, the changes may not have been properly endorsed to the implementers, leading to a lack of awareness regarding the changes in maternal schistosomiasis treatment [14,60].

The WHO had a goal of morbidity control, a prevalence of heavy-intensity infection below 5% in all sentinel sites by 2020 [11]. This brings the question: has the goal of disease control been reached among pregnant women? As the WHO guidelines did not include 95% confidence intervals, the pooled prevalence of *S. haematobium* and *S. mansoni* suggests that disease control has been achieved among pregnant women. However, an upper limit of 95% CI surpassing 5% and the paucity of studies in other endemic countries do not prove this achievement further. It is not easy to compare whether our results mirror the global initiative for schistosomiasis control. In African countries, routine monitoring and evaluation of schistosomiasis disease control are limited to school-age children [61]. This selectivity in monitoring may fail to capture unintentional reservoirs, such as adults, particularly pregnant women.

In 2022, the WHO strongly recommended including pregnant women after the first trimester and lactating women in the treatment coverage in endemic communities where the infection is ≥10%. The annual preventive chemotherapy in this group, aside from those two years and above, includes a single dose of praziquantel at ≥75% treatment coverage to control schistosomiasis morbidity and move forward to achieve the elimination of schistosomiasis as a public health problem. A specific systematic review commissioned by the Guideline Development Group presented data to prove the safety of praziquantel for preventive chemotherapy and treatment in children aged ≥2 years, adults, pregnant women after the first trimester, and lactating women with moderate certainty of evidence [12]. In the same WHO Guideline on Control and Elimination of Human Schistosomiasis in 2022, the WHO proposes that health facilities offer praziquantel treatment to all people positive for schistosomiasis without regard to age, including pregnant women who are infected (but excluding those in their first trimester), lactating women and pre-SAC aged <2 years. To implement this, pregnancy status should be evaluated by subtly asking the woman. If she is unsure of her status, there should be a negative urine-based test before treatment is given.

Schistosomiasis is a highly debilitating but treatable and preventable neglected tropical disease [1]. However, despite the disease burden, there are still insufficient studies and resource allocation to schistosomiasis research and control. One of the barriers to the diagnosis and treatment of schistosomiasis in pregnancy includes poor sensitivity of the Kato–Katz technique in diagnosing schistosomiasis infection. These problems are encompassed by the lack of maternal health care access, research, and policies in the affected countries. More studies need to be conducted to help us determine the actual global burden of schistosomiasis among pregnant women. For example, several African countries, including Liberia, Cote d’Ivoire, Sierra Leone, and Guinea, have a high pediatric schistosomiasis endemicity but have no data for the prevalence of maternal schistosomiasis [4]. There are also no prevalence data of schistosomiasis infection in the Middle East, Indonesia, and China, countries that are also endemic to the infection [62]. There are also limited studies on the maternal and neonatal adverse outcomes caused by schistosomiasis. This data is vital in the clinical management of pregnant patients afflicted with schistosomiasis. This data will also inform health policymakers in improving antenatal care for these patients. Improving the information on schistosomiasis burden among pregnant patients will require standardizing the specimen, time and frequency of collection, and diagnostic tests used to detect schistosomiasis among pregnant patients.

This study provided the most recent and comprehensive global prevalence of schistosomiasis among pregnant patients. This study also showed the prevalence of schistosomiasis in pregnant women based on the intensity of the infection. Only studies that used the WHO classification were included in the analysis [63]. However, the results presented in this meta-analysis and meta-regression may be subject to several limitations. While we tried to identify studies published before and after the approval of praziquantel MDA for pregnant women, most studies did not report whether the population included in their studies received praziquantel. This may have affected the result of prevalence studies. Multivariable meta-regression, which may evaluate the relationship of a single covariate to logit prevalence while adjusting for other covariates, was not performed due to a lack of studies. Egger’s test for asymmetry showed that there was not sufficient evidence of publication bias or small study effects for *S. haematobium* and *S. mansoni*; however, it is essential to note that these methods may be unreliable, especially in cases with studies having low or high prevalence outcomes [64]. Results may also be unreliable in the case of *S. mansoni* where there are fewer than ten studies, thus decreasing the statistical power of the test. While it is unlikely that studies reporting single proportions or prevalence decide against publishing results compared to comparative studies, publication bias may arise from the exclusion of studies published in other languages, and, given the databases searched, exclusion of non-published data possibly due to poorly designed studies, which can either attenuate or accentuate the pooled prevalence.

## 5. Conclusions

The meta-analysis results show a prevailing health problem of schistosomiasis infection during pregnancy in various countries worldwide. It also emphasizes that schistosomiasis disease control has yet to be achieved. Additionally, it highlights the need for integrated prevention and control strategies and sufficient resource allocation for maternal schistosomiasis research to improve its diagnosis and treatment. Furthermore, antenatal care should be implemented especially in areas endemic to schistosomiasis, to understand better the lasting impacts of schistosomiasis infection on pregnant women and their offspring.

## Figures and Tables

**Table 1:** Results of univariable meta-regression analysis exploring the source of heterogeneity for studies on the prevalence of *S. haematobium* in pregnant women.

Variable	Number of Studies Included in Analysis	β (95% CI)	*p*-Value	I^2^ (%)	T^2^
**Study Design**					
Cohort (ref: Cross-Sectional)	21	−0.01 (−1.09, 1.07)	0.9840	98.47	1.13
**Study Conducted after Praziquantel MDA for Pregnant Women**					
Yes (ref: No)	20	0.13 (−0.98, 1.24)	0.8187	98.50	1.17
**Sample Size**					
≥500 (ref: <500)	21	−0.34 (−1.34, 0.66)	0.5076	98.38	1.09
**Specimen for Diagnosis**					
Urine (ref: others)	21	−0.21 (−1.23, 0.81)	0.6836	98.40	1.12

## Data Availability

The data supporting reported results can be found in the Supplementary File.

## Supplementary Materials

The following supporting information can be downloaded at: https://www.mdpi.com/article/10.3390/tropicalmed7110354/s1, File S1: Search Strategy; Table S1: Qualitative synthesis of schistosomiasis studies included in the meta-analyses; Table S2: Quality assessment of cross-sectional studies using the Newcastle–Ottawa Scale; Table S3: Quality assessment of cohort studies using the Newcastle–Ottawa Scale; Table S4: Prevalence of schistosomiasis in pregnant women based on the intensity of infection; Table S5: Egger’s test of publication bias.
