# Supplementary material for: The Global Prevalence of Schistosoma mansoni, S. japonicum, and S. haematobium in Pregnant Women: A Systematic Review and Meta-Analysis
Source: Trop Med Infect Dis. 2022 Nov 4;7(11):354. doi: 10.3390/tropicalmed7110354 (PMC9693339; doi:10.3390/tropicalmed7110354)
Supplement: Supplementary file 1 [file tropicalmed-07-00354-s001.zip › tropicalmed-1957109-supplementary.pdf]

Tropical Medicine and Infectious Disease

MDPI

Supplementary Materials for The Global Prevalence of Schistosoma Mansoni, S. Japonicum, and S. Haematobium in Pregnant Women: A Systematic Review and Meta-Analysis

File S1. Search Strategy

A. Searchstring on OVID

1. exp Schistosomiasis/ or (schistosomiasis or schistosomiases or bilharziases or bilharziasis).mp.
2. (Katayama adj3 fever).mp.
3. (schistoma adj3 infection*).mp.
4. 1 or 2 or 3
5. exp Pregnancy/ or exp Pregnant Women/ or exp Gravidity/ or (pregnan* or gestation or gravidit* or multigravidit* or nulligravidit* or primigravidit* or gravid).mp.
6. 4 and 5
7. limit 6 to english language

Download Date: 9/15/2020

B. Searchstring on Scopus

(schistosomiasis OR bilharziasis OR bilharziases OR "Katayama Fever" OR "Schistosoma Infection") AND (pregnancy OR pregnant OR gestation OR gravidity OR gravid) AND NOT INDEX (medline) AND (LIMIT-TO (SRCTYPE , "j")) AND (LIMIT-TO (DOCTYPE , "ar")) AND (LIMIT-TO (LANGUAGE , "English"))

Download Date: 9/11/2020

C. Searchstring on CINAHL

S3 S1 AND S2
S2 pregnancy OR pregnant OR gestation OR gravidity OR gravid
S1 schistosomiasis OR bilharziasis OR bilharziases OR "Katayama Fever" OR "Schistosoma Infection"
Download Date: 9/15/2020

Table S1. Qualitative synthesis of schistosomiasis studies included in the meta-analyses.

First Author	Year of Publication	Study Design[1]	Study Period	Sample Size	Sample[2]	Method of schistosomiasis diagnosis	Country	Prevalence *S. mansoni*	*S. japonicum*	*S. haematobium*
Agbota	2019	CS	2014-2018	157	U	Urine filtration, centrifugation and microscopy	Benin			0.2357
Anchang-Kimbi	2017	CS	2014	250	U	Urine microscopy and/or urine reagent strip (microhematuria)	Cameroon			0.4680
Tonga	2019	CS	2016	282	U	Urine centrifugation and fecal Kato-Katz and formol-ether followed by microscopy	Cameroon	0.2801		0.0496
Wepnje	2019	CS	2016-2018	368	U	Urine filtration and microscopy	Cameroon			0.2228
Gebreegziabiher	2014	CS	2011-2012	85	S	Kato-Katz	Ethiopia	0.2000		

Flügge	2020	CS	2014-2017	123	U, S	Urine filtration and microscopy	Gabon			0.1138
Adegnika	2010	CO	2003-2004	388	U, S	Urine filtration and microscopy	Gabon			0.1057
Manego	2017	CS	2010-2011	591	U	Urine filtration; microscopy	Gabon			0.0677
Ahenkorah	2020	CS	2013-2014	334	U	Urine sedimentation; microscopy	Ghana	0.0030		0.0030
Tay	2017	CS	2012	375	U, S	Urine membrane filtration technique, and microscopy	Ghana	0.0750		0.0453
Mombo-Ngoma	2017	CO	2009-2013	1115	U	Urine filtration method	Ghana			0.0924
Ondigo	2018	CO	2013-2017	99	S	Kato Katz	Kenya	0.4240		
Malhotra	2015	CO	2006-2009	450	U, S, and plasma	Stool and urine sample for *S. haematobium* ova and ELISA detection of SWAP-specific IgG4 antibodies in plasma	Kenya			0.3244
Kihara	2015	CS	2011	158	U	Nuclear pore filtration and microscopy	Kenya			0.3694
Gallagher	2005	CO	1996-2002	249	U	Urine filtration and microscopy	Kenya			0.0683
McClure	2014	CO	2006-2009	514	U, S	Urine sedimentation, and microscopy	Kenya			0.1710
Thigpen	2011	CS	2002-2004	848	U, S	Urine sedimentation and microscopy, Kato–Katz	Malawi	0.1440		0.3231
Ayoya	2006	CS	2002	131	U	Urine sedimentation; microscopy	Mali			0.2300
Eyo	2012	CS	NA	172	U	Urine filtration, centrifugation, and microscopy	Nigeria			0.2384
Kagu	2007	CS	2005-2006	1040	U	Urine filtration, centrifugation, and microscopy	Nigeria			0.0375
Salawu	2013	CS	2010-2011	313	U	Centrifugation and Microscopy	Nigeria			0.2077
Oyeyemi	2017	CS	2010-2011	261	U	Centrifugation and Microscopy	Nigeria			0.1992
Kurtis	2011	CO	2003-2004	99	S	Kato Katz	Philippines		0.5354	
Khalid	2012	CS	2010	292	S	Formol–ether concentration and Kato–Katz	Sudan	0.1301		

Ajanga	2006	CS	2004	972	S	Kato-Katz	Tanzania	0.6350		
Woodburn	2009	CS	2003-2005	2498	S	Kato Katz	Uganda	0.1830		
Murenjekwa	2019	CS	2012-2015	4437	U	Urine filtration and microscopy	Zimbabwe			0.1062

[1]CS – cross-sectional; CO – cohort.
[2]U – urine; S – stool; U, S – urine and stool; FT – fallopian tube; RB – rectal biopsy.

Table S2. Quality assessment of cross-sectional studies using the Newcastle-Ottawa Scale.

Study	Selection (max of 5 points)	Comparability (max 2 points)	Outcome (max of 3 points)	Total score
Agbota 2019	3	2	3	8
Eyo 2012	3	0	3	6
Khalid 2012	4	0	3	7
Thigpen 2011	4	2	3	9
Woodburn 2009	4	2	3	9
Kagu 2007	4	1	3	8
Gebreegziabiher 2014	4	1	3	8
Flügge 2020	4	1	3	8
Anchang-Kimbi 2017	4	2	3	9
Kihara 2015	2	0	3	5
Tonga 2019	4	2	3	9
Wepnje 2019	4	0	3	7
Salawu 2013	4	2	3	9
Ahenkorah 2020	4	2	3	9
Oyeyemi 2017	4	1	3	8
Mangeo 2017	2	0	2	4
Tay 2017	3	2	3	8
Ayoya 2006	4	2	3	9
Ajanga 2006	4	2	3	9
Kurtis 2011	3	1	3	7

Table S3. Quality assessment of cohort studies using the Newcastle-Ottawa Scale.

Study	Selection (max of 4 points)	Comparability (max 2 points)	Outcome (max of 3 points)	Total score
Adegnika 2010	4	1	3	8
Ondigo 2018	3	1	2	6
Malhotra 2015	1	2	3	6
Mombo-Ngoma 2017	4	1	3	8
Gallagher 2005	3	1	3	7
McClure 2014	3	1	2	6
Murenjekwa 2019	3	2	2	7

Table S4. Prevalence of schistosomiasis in pregnant women based on the intensity of infection.

First Author	Year of Publication	*S. mansoni*			*S. japonicum*		*S. haematobium*		
		Light Infection	Moderate Infection	Heavy Infection	Light Infection	Moderate Infection	Light Infection	Moderate Infection	Heavy Infection
Kurtis	2011				0.42424	0.11111			
Ondigo	2018	0.27273	0.09091	0.06061					

Anchang-Kimbi	2017						0.25600	0.21200
Murenjekwa	2019						0.09580	0.00859
Wepnje	2019						0.16033	0.06250
McClure	2014						0.13362	0.03736
Khalid	2012	0.04452	0.07192	0.01370				
Ajanga	2006	0.45276	0.127	0.05525				

Table S5. Egger's test of publication bias.

Species	Number of Studies	Bias	Standard Error	p-value
S. mansoni	9	–5.14	5.7823	0.40
S. haematobium	21	–0.90	2.9010	0.76
